# Differential Contribution of TRPA1, TRPV4 and TRPM8 to Colonic Nociception in Mice

**DOI:** 10.1371/journal.pone.0128242

**Published:** 2015-07-24

**Authors:** Sonja M. Mueller-Tribbensee, Manoj Karna, Mohammad Khalil, Markus F. Neurath, Peter W. Reeh, Matthias A. Engel

**Affiliations:** 1 Institute of Physiology and Pathophysiology, Friedrich-Alexander-Universität Erlangen-Nürnberg, Erlangen, Germany; 2 Department of Medicine 1, Universitätsklinikum Erlangen, Erlangen, Germany; University of South California, UNITED STATES

## Abstract

**Background:**

Various transient receptor potential (TRP) channels in sensory neurons contribute to the transduction of mechanical stimuli in the colon. Recently, even the cold-sensing menthol receptor TRPM(melastatin)8 was suggested to be involved in murine colonic mechano-nociception.

**Methods:**

To analyze the roles of TRPM8, TRPA1 and TRPV4 in distension-induced colonic nociception and pain, TRP-deficient mice and selective pharmacological blockers in wild-type mice (WT) were used. Visceromotor responses (VMR) to colorectal distension (CRD) in vivo were recorded and distension/pressure-induced CGRP release from the isolated murine colon ex vivo was measured by EIA.

**Results:**

Distension-induced colonic CGRP release was markedly reduced in TRPA1^-/-^ and TRPV4^-/-^ mice at 90/150 mmHg compared to WT. In TRPM8-deficient mice the reduction was only distinct at 150 mmHg. Exposure to selective pharmacological antagonists (HC030031, 100 μM; RN1734, 10 μM; AMTB, 10 μM) showed corresponding effects. The unselective TRP blocker ruthenium red (RR, 10 μM) was as efficient in inhibiting distension-induced CGRP release as the unselective antagonists of mechanogated DEG/ENaC (amiloride, 100 μM) and stretch-activated channels (gadolinium, 50 μM). VMR to CRD revealed prominent deficits over the whole pressure range (up to 90 mmHg) in TRPA1^-/-^ and TRPV4^-/-^ but not TRPM8^-/-^ mice; the drug effects of the TRP antagonists were again highly consistent with the results from mice lacking the respective TRP receptor gene.

**Conclusions:**

TRPA1 and TRPV4 mediate colonic distension pain and CGRP release and appear to govern a wide and congruent dynamic range of distensions. The role of TRPM8 seems to be confined to signaling extreme noxious distension, at least in the healthy colon.

## Introduction

Distension-induced colonic pain during physiological digestive processes is a major problem in gastroenterological practice. The majority of patients who consult a gastroenterologist suffer from pain associated with inflammatory bowel diseases (IBD) or irritable bowel syndrome (IBS) [1,2]. Afferent spinal nerves encode noxious stimuli to the colon, whereas vagal sensory neurons may also play a role in nociception of the proximal gastrointestinal tract. To date it is poorly understood how mechanical force is converted into an electrochemical signal. The existence of a mechanoreceptor signalling complex comprising a cluster of stretch-activated membrane ion channels is assumed [3]. The structural identity of its components is still unknown, however, epithelial sodium channel with degenerin subunits (DEG/ENaC) and various TRP channels have been implicated in mechanotransduction [2–4]. The family of mammalian TRP ion channels comprises six subfamilies with a total of 27 members in humans and 28 in the mouse [2]. TRPV(vanilloid)1 and recently TRPV4, TRPA(ankyrin)1 and TRPM(melastatin)8 were proposed to be involved in pressure/distension-induced mechanoreception or pain in the colon [5–15]. TRPV1 is probably the most extensively studied subtype of the TRP family with regard to somatic but also visceral pain processing. It is activated by noxious heat, low pH and the chili pepper extract capsaicin which causes distinct visceral pain when applied as an enema [4]. Various TRPV1 channel antagonists have even been investigated in several clinical trials, however, caused critical side effects such as hyperthermia [16]. Still, other promising candidates within the TRP family such as TRPV4 are potential targets for the alleviation of abdominal pain. Physiologically, TRPV4 (formerly called OTRPC4, TRP12 or VR-OAC) responds to hypoosmotic stimuli, however, there has been some evidence that TRPV4, expressed in Xenopus oocytes, was also directly activated by membrane stretch in excised patches, excluding the involvement of cytoplasmatic factors in mechanotransduction [17–20]. Accordingly, TRPV4 is proposed to play a major role in colonic high-threshold mechanosensory function as mechanosensory responses were found strongly reduced in TRPV4 knockout mice [4,16]. Another candidate supposedly participating in mediating colonic nociception is TRPA1 [4,6,16]. Its molecular structure comprises a large number of ankyrin repeats which may function as a spring and intracellular anchor transmitting forces to the channel [4,21]. On the other hand, TRPA1 strongly interacts with the cell lipid membrane in which it is embedded [22]. Correspondingly, TRPA1 has been shown to be indirectly activated by compounds such as trinitrophenol and lipopolysaccharides that integrate in and crenate the plasma membrane [23,24]. Among its chemical activators are extracts of mustard, cinnamon, onions, and garlic [4]. However, physiologically more important TRPA1 is also activated by endogenous lipid peroxidation products (LPP) of oxidative stress such as 4-hydroxy-nonenal and acrolein that accumulate during inflammation. LPPs activate the sensitized TRPA1 receptor channel during experimental colitis which leads to increased release of the proinflammmatory neuropeptide substance P initiating and maintaining colitis [25]. Finally, TRPM8 is increasingly recognized for its role in modulation of pain and nociception... The channel was initially demonstrated to be gated in response to cold temperatures and cooling agents such as the peppermint constituent menthol. Its role in injury-evoked cold and mechanical allodynia in the somatic sensory nervous system was recently characterized [12]. Concerning bowel hypersensitivity, peppermint remedies are reported to reduce symptoms, while the underlying molecular mechanisms remain unclear. Both, pro- and anti-nociceptive roles were reported for TRPM8 in high-threshold colonic afferents, as it produces initial activation followed by mechanical desensitization [12]. However, the analgesic effect of TRPM8-expressing afferents may also be centrally mediated through activation of inhibitory interneurons in the spinal dorsal horn [16]. All of these TRP channels, except TRPM8, are enriched within DRG neurons innervating the colon compared to somatic DRGs [2]. The mentioned TRP channels are believed to play an essential role in modulation of a putative mechanoreceptor signaling complex [3,4]. Many previous studies that analyzed TRP channel function in colonic mechanotransduction employed *in vitro* electrophysiology or measured visceromotor responses (VMR) to colorectal distension (CRD), although only one pressure or a narrow pressure range (15–60 mmHg) was applied [5–15]. In the present study we investigated VMR to CRD over a wide range of pressures/distensions *in vivo* and quantified neuropeptide (calcitonin gene-related peptide, CGRP) release from the nerve terminals of the isolated murine colon *ex vivo* as an index of peptidergic nociceptor activation. We compared TRPA1, TRPV4, and TRPM8 knockout mice and used selective pharmacological blockers of each TRP channel to analyze their role in distension-induced colonic nociception and pain. Finally, we compared the efficacy of null mutation and pharmacological TRP antagonists with classical blockers of mechanogated DEG/ENaC and stretch-activated ion channels (SAC), such as amiloride and gadolinium, respectively.

## Materials and Methods

### Animals

Mice of both sexes aged 10–14 weeks were used in these experiments. All experiments were approved by the Animal Protection Authority of the District Government of Mittelfranken, Ansbach, Germany. Breeding pairs of TRPA1^+/-^ mice were donated by Drs. Kwan and Corey (Harvard, Boston, MA) [26]. Mice have been continuously backcrossed to C57BL/6 mice (>9 generations) and were congenic. Homozygous breeding pairs of TRPV4 knockout mice were generously provided by Dr. Liedtke [27], TRPM8 knockouts by Dr. Patapoutian [28]. All mice were genotyped using commercially available primers (Metabion, Martinsried, Germany).

### Solutions and chemicals

Synthetic interstitial fluid (SIF) comprised (in mM) 107.8 NaCl, 26.2 NaCO_3_, 9.64 Na-gluconate, 7.6 sucrose, 5.05 glucose, 3.48 KCl, 1.67 NaH_2_PO_4_, 1.53 CaCl_2_ and 0.69 MgSO_4_, gassed with 95% O2 and 5% CO2 to obtain pH 7.4 [29]. Stock solutions of the TRPA1 antagonist HC030031, the TRPM8 antagonist AMTB hydrochloride and the TRPV4 antagonist RN 1734 (all purchased from Tocris Biosciences, Ellisville, USA) were made up in absolute methanol. Gadolinium(III) chloride, amiloride hydrochloride hydrate and ruthenium red (all purchased from Sigma-Aldrich, Steinheim, Germany) and the final dilutions were prepared by dissolving the stock solutions in SIF on the day of each experiment. The pH was adjusted to 7.4 by adding drops of HCl or NaOH.

### Calcitonin gene-related peptide (CGRP) release measurements

CGRP release measurements from the isolated mouse colon were performed as established before [30]. In the present study, four centimeters of the mouse distal colon were excised, starting at the junction of the sigmoid/rectum. Colons were emptied from stool, rinsed with saline and ligated at one end with a cotton wool string. On the other end a thin flexible tube was inserted and the colon was fastened over the tube (colon serosal side up) so that air could be inflated to produce pressure inside the colon (mucosal side). For equilibration the colon was placed in SIF inside a temperature-controlled shaking bath at 38°C for 30 min. A series of 4 test tubes were each filled with 800μl of SIF and placed in the shaking bath. The fixed colonic tissue was transferred into the first tube (S1). After 5 min the colon was moved into the second tube for another 5 min (S2). In the third tube (S3) the colon was insufflated with air of a defined pressure (between 30 and 150 mmHg) for 5 min. In some experiments the third tube was filled with SIF containing the respective TRPA1, TRPV4 or TRPM8 receptor antagonist or ruthenium red, amiloride, and gadolinium. Finally, the air was drained off and the colon transferred to the fourth tube (S4) for further 5 minutes. Acquired data are shown raw and/or normalized to the S2 baseline value (ΔCGRP release pg/ml eluate).

### CGRP enzyme immunoassay (EIA)

CGRP release was quantified immediately after the end of each experiment using a commercially available enzyme immunoassay (Bertin Pharma, Montigny le Bretonneux, France). At the end of each five minutes exposure period, 100 μl of eluate was mixed with 25 μl of CGRP EIA buffer comprising potassium phosphate buffer (0.1 M), NaCl (0.15 M), 0.1% BSA (g/g), 0.01% sodium azide (g/g) and protease inhibitors. All EIA plates were determined photometrically using a microplate reader (Dynatech, Guernsey, Channel Islands, UK). The detection limit for the CGRP-EIA was 5 pg/ml.

### Measurement and analysis of visceromotor responses (VMR) to colorectal distension (CRD)

Assessment of colon sensitivity in response to CRD was performed as previously published [31]. Electrodes were made by stripping 3 mm of insulation from the ends of teflon-coated stainless steel wires (Science Products GmbH, Hofheim, Germany). 4 days before CRD measurements the electrodes were implanted into the abdominal musculature of mice. Tunneling of the electrodes to the dorsum of the neck was necessary to avoid cropping. During the procedure mice were anesthetized with isoflurane, additionally, metamizole (200 mg/kg BW) was given intravenously continuously during surgery and for 48 hours postsurgery via i.p. injections three times per day. During the recovery period of 4 days, mice were acclimatized to the recording chamber. On the day of testing, mice were lightly anesthetized with isoflurane and restrained in a custom-made clear polycarbonate tube and a balloon catheter (Cordis Power Flex P3; Cordis, Waterloo, Belgium) was smoothly inserted transanally. The tip of the catheter was placed 5 cm from the anus, secured by tape to the tail. Graded distension pressures were applied up to 90 mmHg and each pressure stimulus was applied for 20 s followed by a 4 min recovery period each (inflation time 5s). The various TRP-receptor antagonists (150μl/injection) were injected i.p. 10 minutes prior to the CRD experiments.

Upon distension of the colon a polysynaptic reflex leads to abdominal contractions, comparable to the muscular defense or guarding of peritonitis patients. These visceromotor responses (VMR) to colorectal distension were quantified by measuring the electromyogramm (EMG) of the abdominal musculature (external oblique muscles). EMG activity was amplified and filtered using the Bridge Amp ML221 amplifier, data was recorded, integrated, and analyzed on- and offline using LabChart (both ADInstruments, Colorado Springs, USA). Representative raw EMG recordings are depicted in mV [ordinate: VMR (EMG in mV)]. The integrated electromyogramm (iEMG) values are depicted over the entire pressure range (ordinate: VMR iEMG in μVxs). iEMG baseline activity during the 10 seconds before stimulation was subtracted from the first 10 seconds of the reflex response. At the end of each experiment mice were killed in a 100% carbon dioxide atmosphere by cervical dislocation.

### Statistical analysis

Results are presented as mean ± standard error of the mean (SEM). The number (*n*) that is quoted throughout the manuscript refers to the number of animals used per experiment. The statistical level of significance (P < 0.05, P <0.01) is indicated with one or two asterisks, respectively. Applied statistical tests are denoted in the results section or in the figure legends.

## Results

### TRPA1, TRPV4 and TRPM8 antagonism reduces distension-induced colonic CGRP release

**Fig 1:**
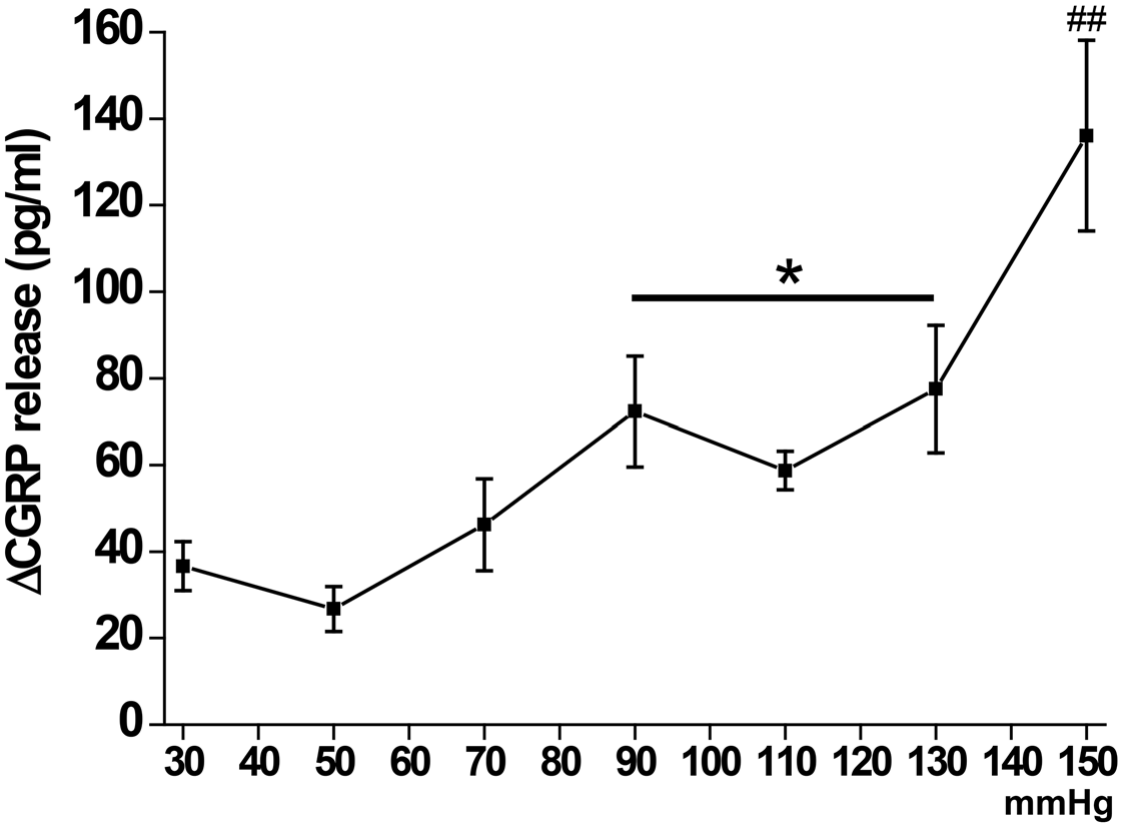
Normalized pressure/distension (30 mmHg—150 mmHg)-induced CGRP release from isolated colon of wild-type C57BL/6 mice (ΔCGRP release in pg/ml). Pressure-dependent increased CGRP release shows a plateau in the middle pressure range. “*” indicates significance level at p < 0.05, “##” at p < 0.01 (Mann-Whitney U-test) versus 30 mmHg-induced colonic CGRP release. Each data point is representative for 6–10 experiments with different animals.

**Fig 2:**
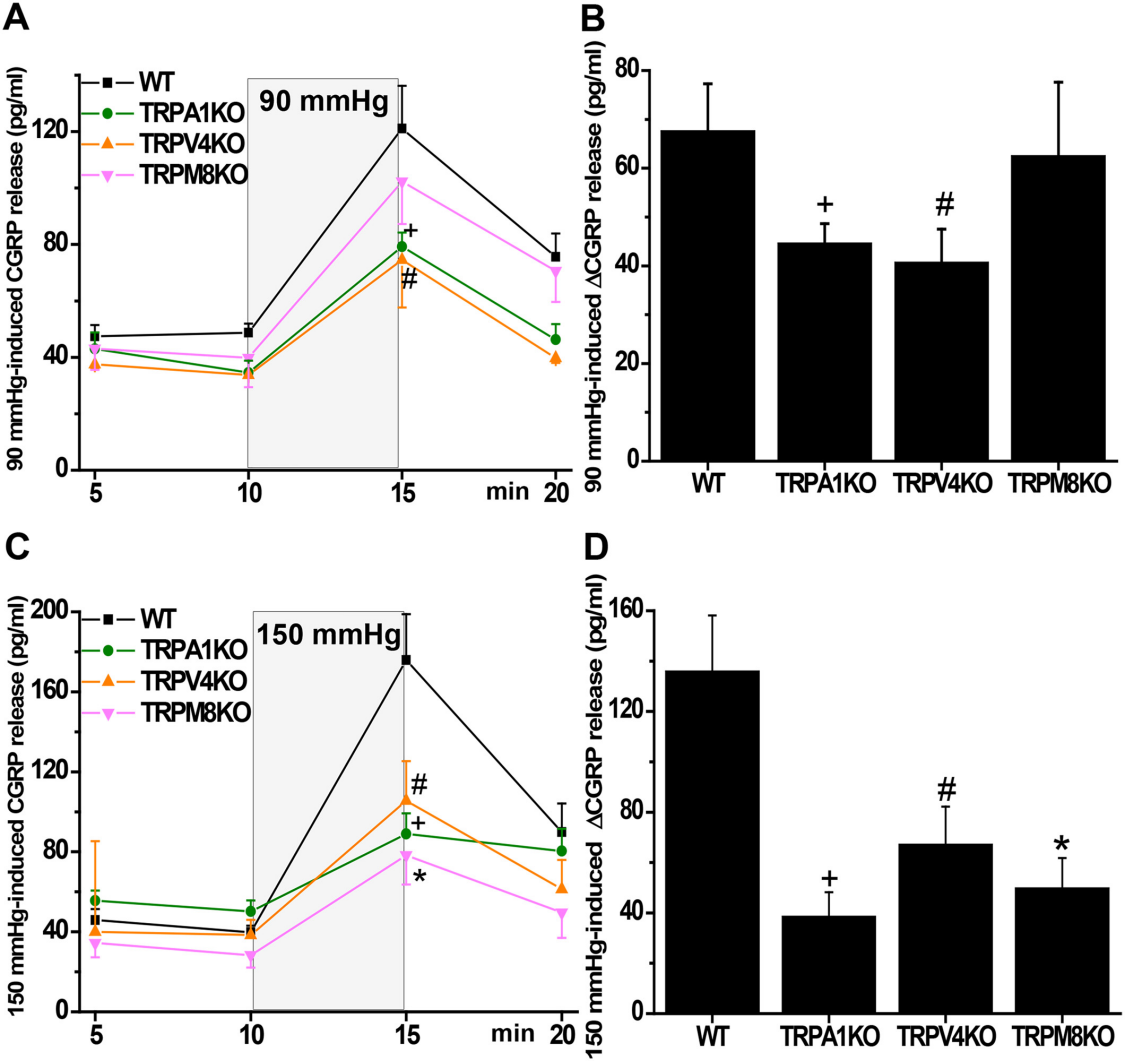
(A,C) Time course of 90 and 150 mmHg-induced CGRP release from the isolated colon of wild-type (WT) mice as compared to TRPA1, TRPV4 and TRPM8 knockout mice. (B,D) Normalized 90 and 150 mmHg-induced CGRP release from the isolated colon of WT as compared to TRPA1, TRPV4 and TRPM8 knockout mice. TRPA1^-/-^ and TRPV4^-/-^ mice show reduced pressure-induced colonic CGRP release compared to WTs at both pressure levels. In TRPM8-deficient mice pressure-induced CGRP release was only reduced at the high (150 mmHg) pressure level compared to WT. “+”, “#” and “*” indicate significance levels at p < 0.05 (Mann-Whitney U-test) versus WT mice. Each data point n = 6–10.

In order to analyze whether the lack of either TRPA1, TRPV4 and TRPM8 or respective pharmacological blocks reduce nociceptor activity, we employed the established *ex vivo* model of measuring pressure/distension-induced CGRP release from the isolated mouse colon [30]. Initial experiments in wild-type (WT) mice demonstrated a bimodal pressure-response relationship with a plateau between 90 mmHg and 130 mmHg (Fig 1). For further CGRP release experiments we used middle (90 mmHg) and high (150 mmHg) pressure to characterize the different mouse phenotypes. Baseline CGRP release from the isolated colon of WT and the different knockout mice did not significantly differ (Fig 2). At 90 mmHg colonic CGRP release from WT mice increased by ~70 pg/ml, and that from TRPM8-deficient mice was not significantly less. However, colon from TRPA1-deficient mice showed only ~45 pg/ml increase, and CGRP release from TRPV4^-/-^ colon was about halved compared to WT (Fig 2A and 2B). 150 mmHg-induced CGRP release in WT mice was substantially increased compared to 90 mmHg (Fig 2C and 2D). Colon from TRPA1 knockout mice showed about the same amount of CGRP release as with 90 mmHg, however, relative to WT the deficit was much more prominent at 150 mmHg. Attenuation of 150 mmHg-induced CGRP release from TRPV4^-/-^ colon was in the range of TRPA1^-/-^ colons. However, in contrast to the middle pressure level, colon from TRPM8-deficient mice showed a distinct reduction of CGRP release to about 60% compared to WT mice.

**Fig 3:**
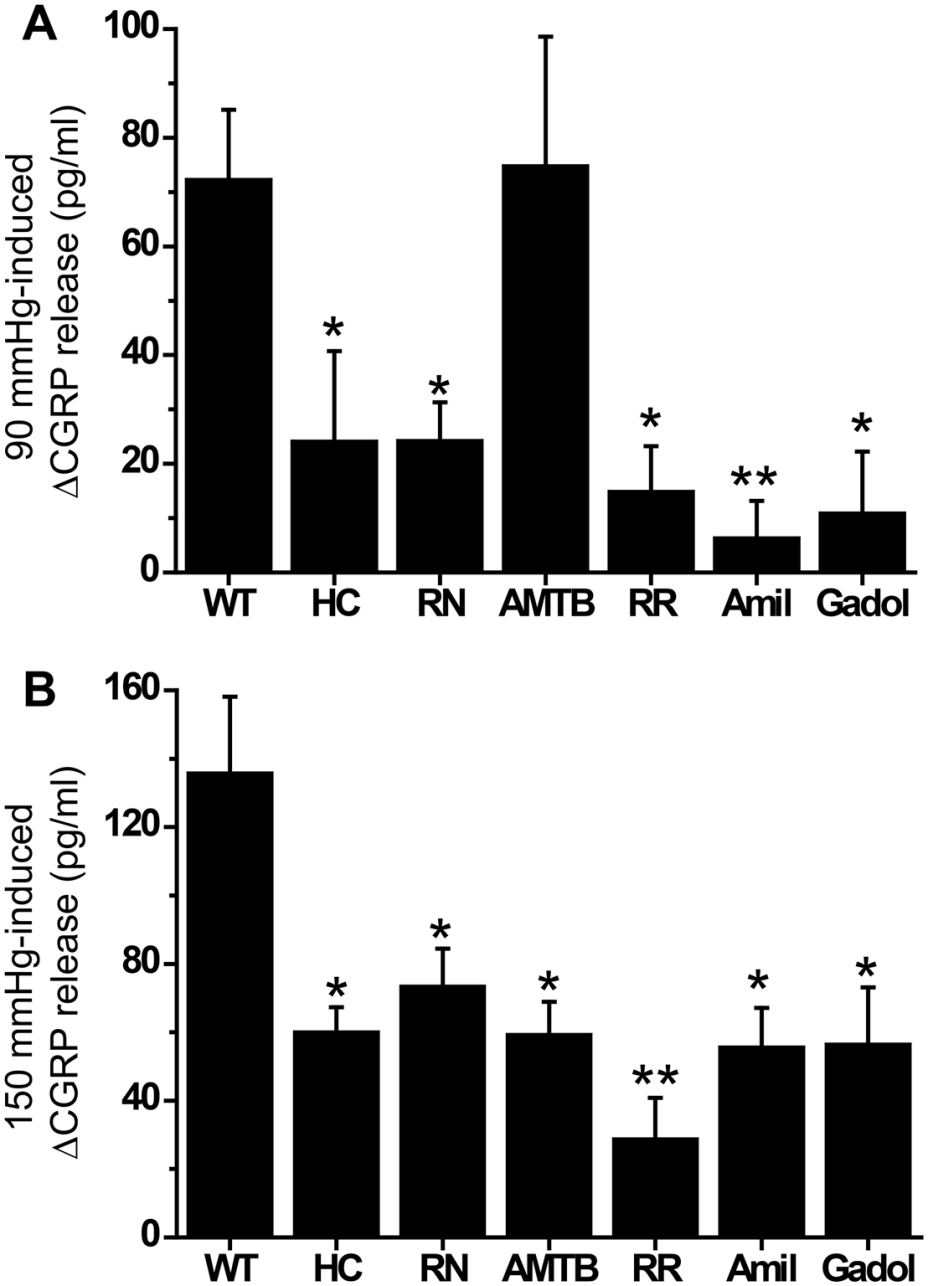
(A) Exposure of the isolated colon of wild-type (WT) mice to selective antagonists of TRPA1 (HC030031, HC, 100 μM) and TRPV4 (RN1734, RN, 10 μM) inhibited 90 mmHg-induced CGRP release. The TRPM8 antagonist AMTB (10 μM) had no effect at this pressure level. The unselective TRP-channel blocker ruthenium red (RR, 10 μM), the DEG/ENaC antagonist amiloride (Amil, 100 μM) and the SAC blocker gadolinium (Gadol, 50 μM) were about equally effective as HC and RN. (B) Analogeous results at 150 mmHg with the same pharmacological blockers, however, the TRPM8 antagonist AMTB distinctly reduced 150 mmHg-induced CGRP release. “*” indicates significance level at p < 0.05 and “**” at p < 0.01 (Mann-Whitney U-test) versus WT. Each data point is representative for 6–10 experiments.

Consistently, the use of selective pharmacological blockers for each ion channel confirmed the findings gained from the knockouts (Fig 3). The inhibitory efficacy of TRPA1 and TRPV4 antagonists (HC030031, 100 μM; RN1734, 10 μM) was about equal. The TRPM8 receptor antagonist AMTB (10 μM) inhibited neuropeptide release only at the high pressure level, consistent with the findings from the knockouts (Fig 3B). The unselective TRP channel blocker ruthenium red (RR, 10 μM) showed about the same efficacy as the specific TRP channel antagonists (except AMTB at 90 mmHg). Also amiloride (100 μM) and gadolinium (50 μM), the blockers of DEG/ENaC and SACs, appeared equally effective at both pressures compared to RR and the TRP inhibitors (Fig 3).

### Lack of TRPA1, TRPV4 but not TRPM8 function attenuates VMR to CRD up to 90 mmHg

**Fig 4:**
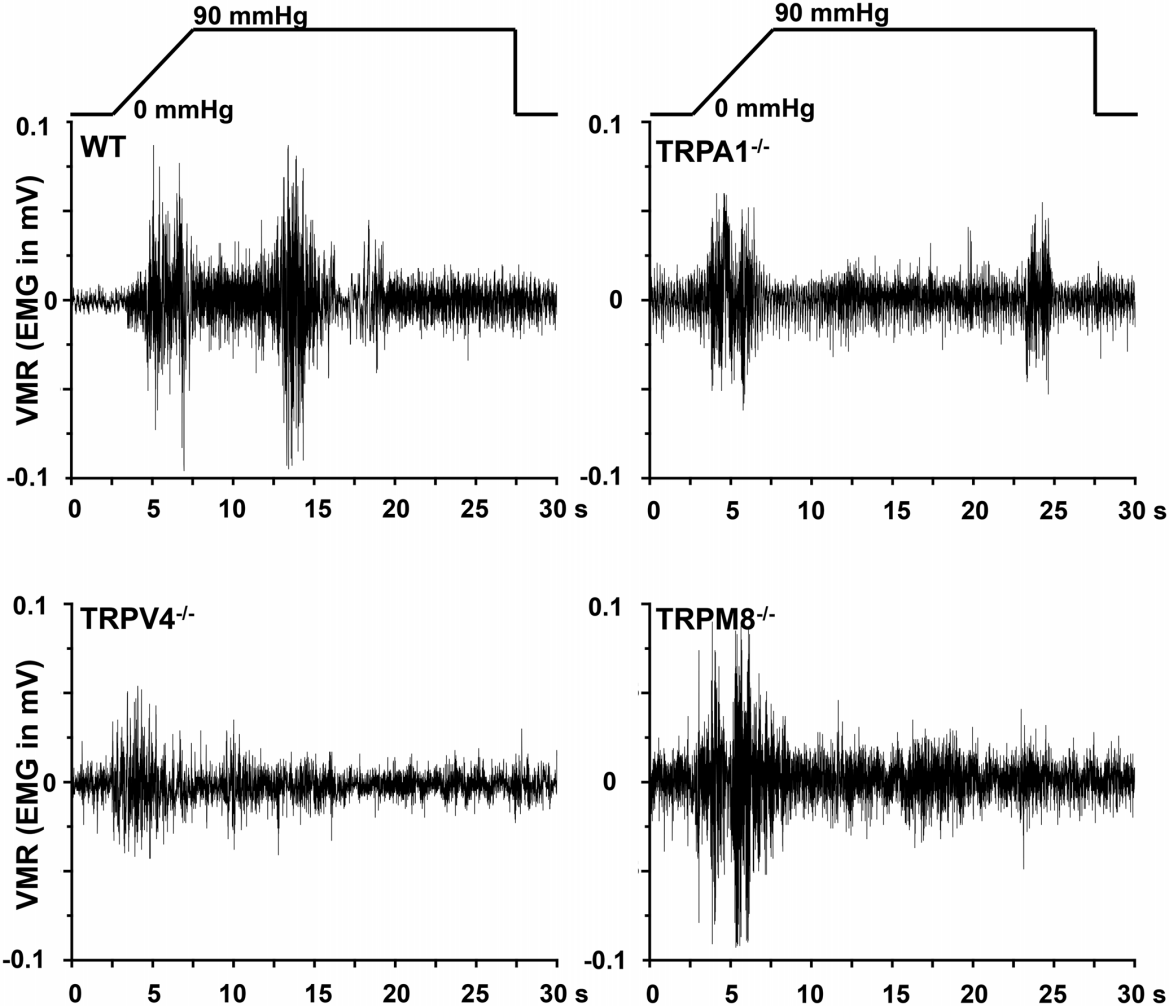
Representative pictures of murine visceromotor responses (VMR) to 90 mmHg-induced colorectal distension. Original EMG (in mV) recorded from the abdominal muscle in the different mouse strains during the 25 s of pressure/distension stimulation.

**Fig 5:**
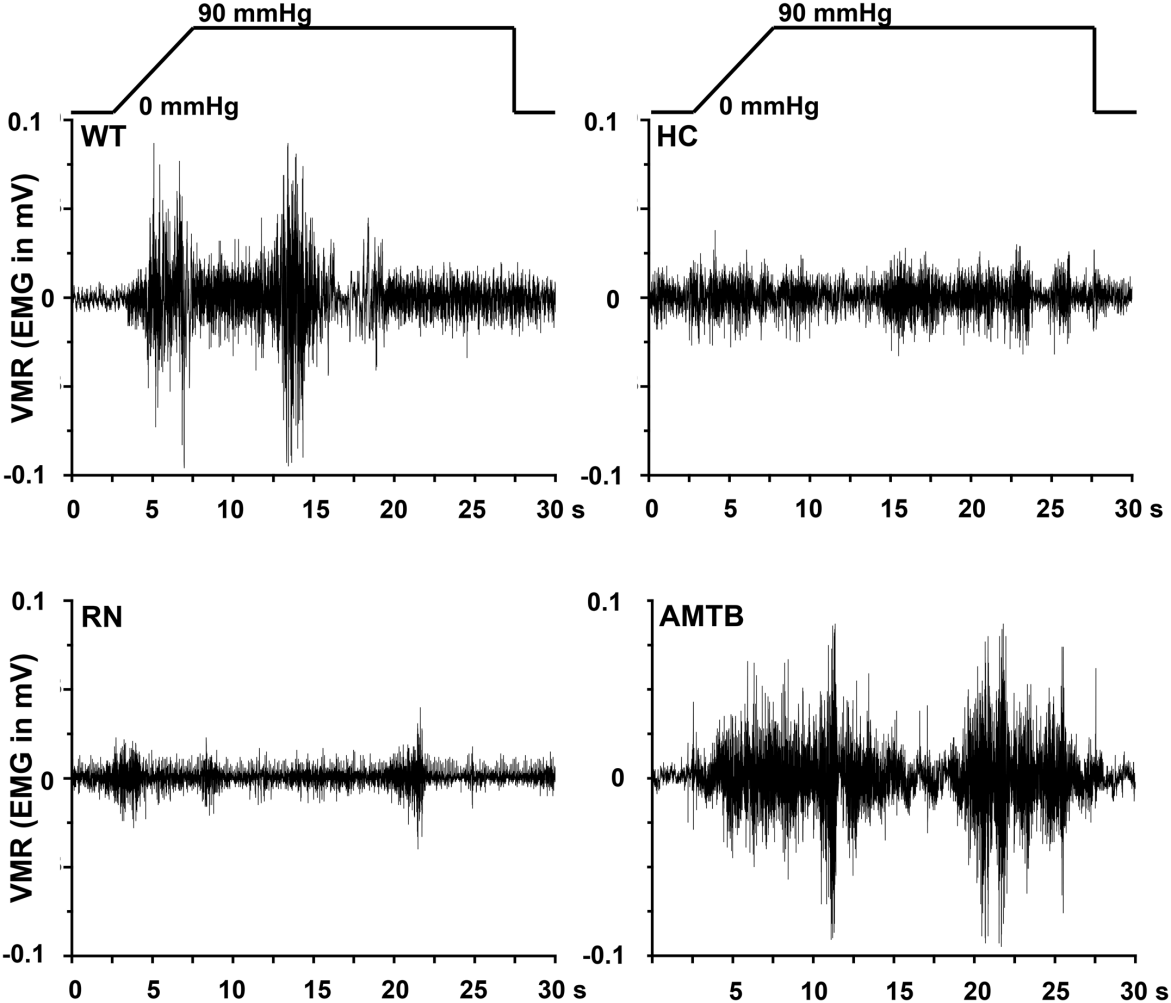
Representative pictures of murine visceromotor responses (VMR) to 90 mmHg-induced colorectal distension. Original abdominal EMG (in mV) in mice treated with the respective TRP receptor antagonist compared to wild-type mice.

**Fig 6:**
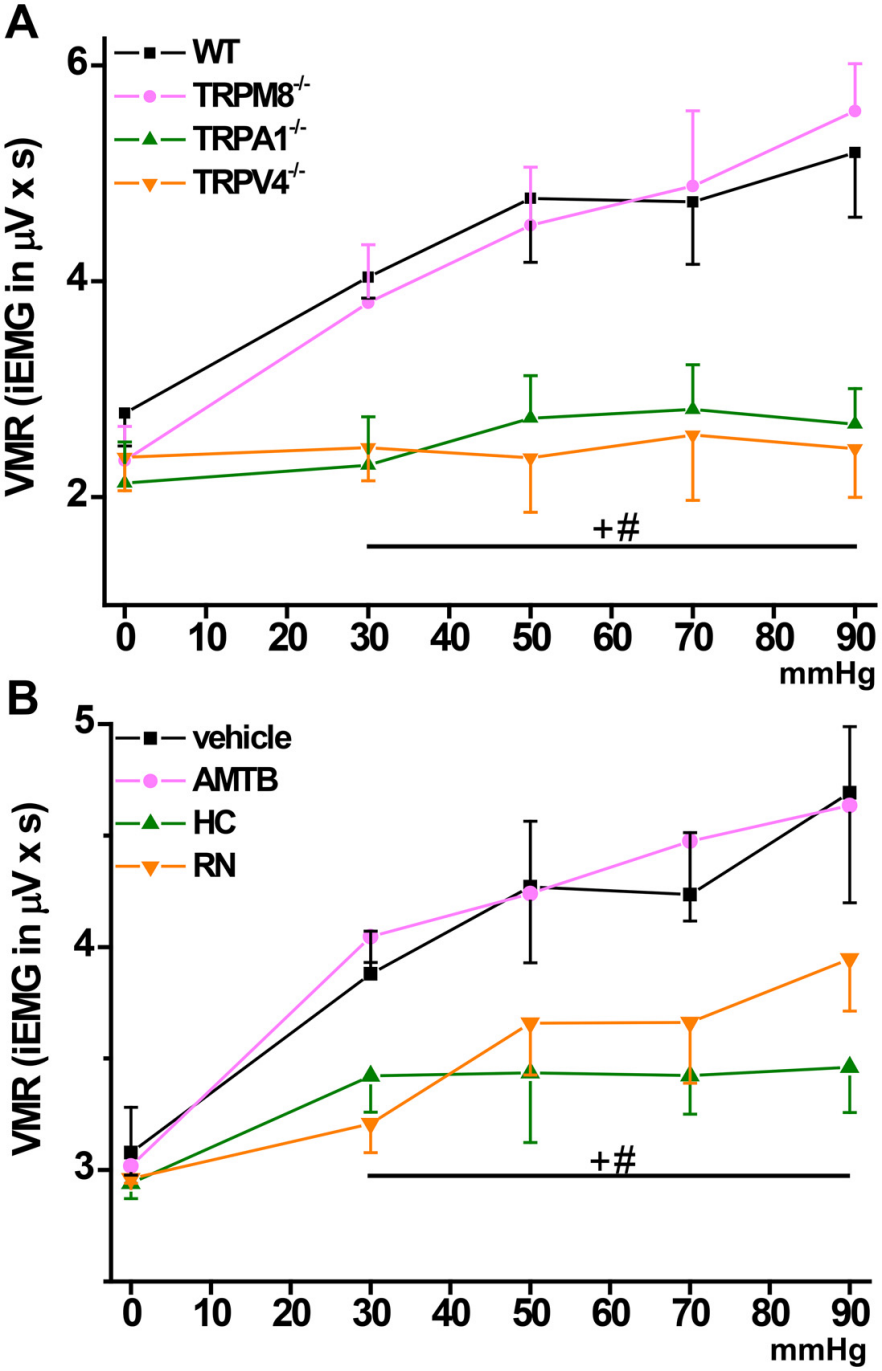
(A) Visceromotor responses to colorectal distension in WT compared to TRPM8, TRPA1 and TRPV4 knockout mice. Visceromotor responses (VMR) are presented as integrated EMG (iEMG in μVxs). TRPA1 and TRPV4 but not TRPM8 knockouts demonstrate reduced pain behavior at all tested pressure levels. Baseline EMG activity was about equal in all mouse strains. (B) Accordingly, the respective pharmacological blockers for TRPA1 and TRPV4 but not TRPM8 reduced VMRs at all pressure levels at the given dosage (HC 10 mg/kg, RN 1 mg/kg, AMTB 10 mg/kg). “+”indicates significance for TRPA1^-/-^ or HC vs. WT, “#” for TRPV4^-/-^ or RN vs. WT (p < 0.05; 2-way ANOVA followed by post hoc LSD test). Each data point is representative for 6–10 experiments.

To scrutinize whether reduced colonic nociceptor activity as a result of TRPA1, TRPV4 or TRPM8 null mutation/block was relevant for distension-induced colonic pain behavior, we chose the established model of measuring VMR to CRD. Integrated EMG activity from the abdominal muscles was measured to quantify the VMR. Baseline EMG-activity of WT and all knockout strains was about equal. The striking differences in EMG activity (in mV) between the different mouse strains are illustrated in Figs 4 and 5 showing representative EMGs at 90 mmHg. VMR to CRD was markedly reduced in TRPA1 and TRPV4 but not TRPM8 knockout mice at all pressure levels up to 90 mmHg (Fig 6A). Consistently, i.p. injection into WT mice of the selective TRPA1 antagonist HC030031 (10 mg/kg BW) or the TRPV4 antagonist RN1734 (1 mg/kg BW) led to reduced VMR which effect was relatively more prominent at the higher pressure levels (Fig 6B). The TRPM8 receptor antagonist AMTB (10 mg/kg BW) did not change VMR to CRD compared to vehicle-injected mice in the tested pressure range. Both, *in vitro* and in *in vivo* experiments did not show sex-specificity of any of the observed responses.

## Discussion

This study provides evidence for a governing role of TRPA1 and TRPV4 in mechanoreception and pain of the healthy mouse colon, whereas TRPM8 is involved in mediating only high-pressure noxious mechanical stimuli.

### TRPA1

Previous electrophysiological single-fiber recordings from vagal and pelvic nerve branches showed that TRPA1-deficient mice exhibit reduced mechanosensitivity of colonic mucosal afferents. TRPA1 deletion led to increased mechanosensory thresholds of high-threshold colonic splanchnic and pelvic serosal as well as mesenteric afferents. Previously, TRPA1^-/-^ mice showed reduced pain behavior in the model of CRD at 80 mmHg colonic pressure but no phenotype in the range of 15 mmHg—60 mmHg [2,6,8]. Our data demonstrate attenuation of CRD-induced VMR throughout a wide pressure range in TRPA1 knockout mice. The distinct attenuation of stimulated colonic CGRP release in TRPA1^-/-^ mice and in WTs treated with HC030031 supports a major role of TRPA1 and peptidergic neurons in colonic mechanotransduction, although a specific working mechanism cannot be demonstrated yet. TRPA1 is known as an almost universal chemoreceptor-channel that is also activated by endogenous substances resulting from oxidative/nitrosative stress and involved in pathomechanisms of chronic inflammatory diseases [24,32,33]. Arachidonic acid is among the intracellular activators of TRPA1 [32] and is an inflammatory mediator in its own right as well as a substrate for the formation of multiple eicosanoids including prostaglandin E_2_ (PGE_2_). Intriguingly, massive amounts of PGE_2_ are formed and released from the isolated colon upon distension, and the stimulus-response relationship (PGE_2_ versus pressure) is even steeper than for distension-induced CGRP/substance P release [30]. In fact, the pancellular phospholipase A2, releasing arachidonic acid from plasma and endosomal membranes, is definitely mechanosensitive and activated by membrane stretch [34–36]. This could be a candidate mechanism involving TRPA1 in mechanosensitivity, although the receptor-channel itself is not stretch-activated.

### TRPV4

Alike TRPA1 and TRPV1, TRPV4 appears to function as a polymodal molecular integrator of various microenvironmental stimuli. TRPV4 was initially described as the transducer of hypoosmotic stimuli [27]. It was also characterized as a mechanosensor since the channel opens in response to hypotonicity-induced cell swelling and shear stress [37]. The physiological relevance of a TRPV4 homolog as an osmotic and mechanical sensor was demonstrated in an elegant *in vivo* approach assessing avoidance behaviour towards pertinent stimuli in transgenic Caenorhabditis elegans [38]. Reduced pain behavior upon noxious somatic mechanical stimuli was reported in TRPV4^-/-^ mice [27,39,40]. Functional significance of the TRPV4 receptor in colonic DRGs was first derived from pharmacological studies using the non-selective TRPV4 agonist 4α-PDD to induce calcium transients and 5,6-EET formation which potentiated mechanosensory responses in WT but not TRPV4^-/-^ mice [2,7,9]. The argument for arachidonic acid possibly mediating secondary mechanosensitivity of TRPA1 (see above) also applies to TRPV4, because this precursor of all eicosanoids, including 5,6-EET, is among the endogenous TRPV4 agonists [39,41,42]. Recently, pain responses to colorectal distension, studied at 80 mmHg, were found reduced to half in TRPV4^-/-^ mice [7]; we demonstrate an even larger deficit. In addition, similar to TRPA1^-/-^ mice, we observed a distinct attenuation of VMR over the entire pressure range in TRPV4^-/-^ mice. The consistent influence of TRPV4 null mutation and pharmacological block on distension-induced colonic CGRP release indicate a peripheral site of action.

### TRPM8

Previous work addressing TRPM8 in the mouse colon found immunoreactive expression in a subpopulation of spinal sensory afferents and an extensive co-expression with CGRP in the serosa, in myenteric ganglia and at the base of the mucosa [12,41,42]. The data on pro- or anti-nociceptive effects of TRPM8 are confusing at first glance. Recently, exposure to the TRPM8 agonist icilin left the mechanoreceptive colonic nerve fibers with a slight transient desensitization (to punctuate pressure) that was interpreted as an indirect, secondary, effect on TRPV1 and/or TRPA1 [12]. Such a cross-desensitization may also account for a recently demonstrated inhibition of capsaicin (TRPV1)-induced CGRP release by icilin in an *ex vivo* model of the distal colon [43]. Pharmacological approaches often bear the burden of unselectivity. Icilin was found to not only activate TRPM8 but also TRPA1 and it was shown to even inhibit TRPM8 through mechanisms different from desensitization that do not require the prototypical interaction site in the S2-S3 loop [44–48]. In our model, using the same anatomical region (distal colon), both pharmacological block and TRPM8 null mutation inhibited high-pressure mechanically induced CGRP release which indicates a pronociceptive role for TRPM8. In agreement, a recent study supports a pronociceptive role of TRPM8 in colitis-induced visceral hyperalgesia in mice [42]. Enemas with the TRPM8 agonist WS-12 induced behavioral visceral pain-like responses which were much greater in mice with colitis compared to controls and were decreased by pretreatment with the TRPM8 antagonist AMTB [42]. Summarized, these collective data suggest that TRPM8 plays an important role in colonic nociception. Whether TRPM8 modulation results in pro- or anti-nociception may depend on the noxious stimulus (physical, chemical, thermal), on the concentration of the pharmacological agonist (TRPM8 sensitization vs. desensitization) and on the state of the TRPM8 receptor in healthy or inflamed tissue (normal vs. sensitized vs. desensitized) which may explain contradictions between different studies. In somatosensory neurons TRPM8 serves as a specific transducer channel for cold stimuli, a mechanism that would involve it in mechanonociception is not known [41]. On a higher level of nociceptive processing, in the spinal dorsal horn, input from TRPM8-expressing cutaneous primary afferents (“cold fibers”) seems to exert antinociceptive, gate-controlling, effects in an animal model of neuropathic pain [49,50]. Accordingly, a recent paper demonstrated that TRPM8 is essential for mediating menthol’s analgesic effects in vivo in various models of acute and inflammatory pain [51]. Genetic deletion and pharmacological inhibition of TRPM8 would, thus, lead to disinhibition of the spinal “gate control” mechanism and result in increased, not diminished, pain responses. However, the marked attenuation of high-pressure stimulated CGRP release in this study rather suggests a peripheral pronociceptive role of TRPM8. Further *in vivo* studies in humans have to analyze peak pressure levels in the GI tract during digestive processes in the healthy state and under pathologic circumstances. Due to ethical restrictions high pressure CRD could not be performed in mice. However, it seems likely that TRPM8 is a promising new target for pain relief in patients with IBD and IBS for two reasons. First, our *in vitro* studies reveal that the TRPM8 antagonist AMTB does inhibit nociceptor activity (CGRP release) at high pressures to about the same degree as TRPA1 and TRPV4 antagonists did. Indeed, high-amplitude propagating contractions (HAPCs) in the human colon were shown to reach about 160 mmHg [52]. Second, inflammatory processes (IBD) or post-inflammatory (IBS) conditions/states of nociceptor sensitization decrease mechanical thresholds, a mechanism that may ascribe TRPM8 an important role under pathologic conditions.

### DEG/ENaC and SAC

Amiloride is an antagonist of DEG/ENaC and ASIC channels and gadolinium unselectively blocks SACs. However, previous publications identify both compounds to also block various TRP channels, thus, both compounds are unable to discriminate between DEG/ENaC, SAC and TRP-mediated effects [3,53]. In our hands both drugs reduced pressure/distension-induced CGRP release to about the same degree and similar to the unselective TRP channel blocker ruthenium red.

Noteworthy, our study does not present data that indicate that the studied TRP channels are mechanically activated. Additionally, TRP channels are also expressed in other neuronal subpopulations than extrinsic sensory neurons and even in non-neuronal tissue which may both have indirect effects on our results. For example, functional TRPA1 expression was recently demonstrated in intrinsic inhibitory motorneurons, mediating inhibition of spontaneous neurogenic contractions and transit of the colon [54]. Additionally, TRPV4 receptor expression in colonic epithelial cells was reported to participate in intestinal inflammation through the release of various cytokines and chemokines [55]. Our results may, thus, be influenced by the effects of global, genetic null mutation or pharmacological blockage of TRP channels expressed in cells other than extrinsic sensory nerve fibers. Finally, in this study we used the unselective TRP-channel blocker ruthenium red which is also an antagonist of murine Piezo 1 channel, which was recently identified to be mechanically-activated [56]. Both mechano-activated Piezo channels 1 and 2 have not yet been studied in the context of colonic nociception, although a Piezo-blocking peptide GsMTx4, a tarantula venom ingredient, is already available.

### Conclusion

Our results suggest that TRP receptor-channels play a dominant role in colonic mechanonociception and release of pro-inflammatory neuropeptides, and that blockage of TRPA1, TRPV4 and possibly TRPM8 may represent a therapeutic option for patients with colonic discomfort and pain. Especially luminal/topical application of the respective TRP receptor antagonists avoiding systemic side effects may represent an interesting novel approach to these patients. The fact that the efficacy of either TRPA1, TRPV4 or TRPM8 (at high pressure only) block/deletion is about the same suggests that the TRP channels form a redundant modulating system within the mechano-gated receptor complex. Further studies have to clarify the role of the investigated TRP channels in visceral hyperalgesia in (post-) inflammatory states such as IBD or IBS.

## References

[1] Choung RS, Locke GR 3rd. Epidemiology of IBS. Gastroenterol Clin North Am. 2011;40:1–10. 21333897 10.1016/j.gtc.2010.12.006

[2] Holzer P. Transient receptor potential (TRP) channels as drug targets for diseases of the digestive system. Pharmacol Ther. 2011;131:142–70. 10.1016/j.pharmthera.2011.03.006 21420431 PMC3107431

[3] Hamill OP, McBride DW Jr. The pharmacology of mechanogated membrane ion channels. Pharmacol Rev. 1996;48:231–52. 8804105

[4] Brierley SM. Molecular basis of mechanosensitivity. Auton Neurosci. 2010;153:58–68. 10.1016/j.autneu.2009.07.017 19683967

[5] Alessandri-Haber N, Dina OA, Joseph EK, Reichling DB, Levine JD. Interaction of transient receptor potential vanilloid 4, integrin, and SRC tyrosine kinase in mechanical hyperalgesia. J Neurosci. 2008;28:1046–57. 10.1523/JNEUROSCI.4497-07.2008 18234883 PMC6671413

[6] Brierley SM, Hughes PA, Page AJ, Kwan KY, Martin CM, O'Donnell TA., et al The ion channel TRPA1 is required for normal mechanosensation and is modulated by algesic stimuli. Gastroenterology. 2009;137:2084–95. 10.1053/j.gastro.2009.07.048 19632231 PMC2789877

[7] Brierley SM, Page AJ, Hughes PA, Adam B, Liebregts T, Cooper NJ., et al Selective role for TRPV4 ion channels in visceral sensory pathways. Gastroenterology. 2008;134:2059–69. 10.1053/j.gastro.2008.01.074 18343379 PMC2504007

[8] Cattaruzza F, Spreadbury I, Miranda-Morales M, Grady EF, Vanner S, Bunnett NW. Transient receptor potential ankyrin-1 has a major role in mediating visceral pain in mice. Am J Physiol Gastrointest Liver Physiol. 2010;298:81–91. 10.1152/ajpgi.00221.2009 PMC2806099 19875705

[9] Cenac N, Altier C, Chapman K, Liedtke W, Zamponi G, Vergnolle N. Transient receptor potential vanilloid-4 has a major role in visceral hypersensitivity symptoms. Gastroenterology. 2008;135:937–46. 10.1053/j.gastro.2008.05.024 18565335

[10] Chan CL, Facer P, Davis JB, Smith GD, Egerton J, Bountra C, et al Sensory fibres expressing capsaicin receptor TRPV1 in patients with rectal hypersensitivity and faecal urgency. Lancet. 2003;361:385–91. 12573376 10.1016/s0140-6736(03)12392-6

[11] Gonlachanvit S, Mahayosnond A, Kullavanijaya P. Effects of chili on postprandial gastrointestinal symptoms in diarrhoea predominant irritable bowel syndrome: evidence for capsaicin-sensitive visceral nociception hypersensitivity. Neurogastroenterol Motil. 2009;21:23–32. 10.1111/j.1365-2982.2008.01167.x 18647268

[12] Harrington AM, Hughes PA, Martin CM, Yang J, Castro J, Isaacs NJ, et al A novel role for TRPM8 in visceral afferent function. Pain. 2011;152:1459–68. 10.1016/j.pain.2011.01.027 21489690

[13] Jones RC 3rd, Xu L, Gebhart GF. The mechanosensitivity of mouse colon afferent fibers and their sensitization by inflammatory mediators require transient receptor potential vanilloid 1 and acid-sensing ion channel 3. J Neurosci. 2005; 25:10981–9. 16306411 10.1523/JNEUROSCI.0703-05.2005 PMC6725875

[14] Jones RC 3rd, Otsuka E, Wagstrom E, Jensen CS, Price MP, Gebhart GF. Short-term sensitization of colon mechanoreceptors is associated with long-term hypersensitivity to colon distention in the mouse. Gastroenterology. 2007;133:184–94. 17553498 10.1053/j.gastro.2007.04.042

[15] Sipe WE, Brierley SM, Martin CM, Phillis BD, Cruz FB, Grady EF, et al Transient receptor potential vanilloid 4 mediates protease activated receptor 2-induced sensitization of colonic afferent nerves and visceral hyperalgesia. Am J Physiol Gastrointest Liver Physiol. 2008;294:1288–98. 10.1152/ajpgi.00002.2008 18325985

[16] Blackshaw LA, Brierley SM, Hughes PA. TRP channels: new targets for visceral pain. Gut. 2010;59:126–35. 10.1136/gut.2009.179523 20007960

[17] Loukin S, Zhou X, Su Z, Saimi Y, Kung C. Wild-type and brachyolmia-causing mutant TRPV4 channels respond directly to stretch force. J Biol Chem. 2010;285:27176–81. 10.1074/jbc.M110.143370 20605796 PMC2930716

[18] Liedtke W, Choe Y, Martí-Renom MA, Bell AM, Denis CS, Sali A, et al Vanilloid receptor-related osmotically activated channel (VR-OAC), a candidate vertebrate osmoreceptor. Cell. 2000;103:525–35. 11081638 10.1016/s0092-8674(00)00143-4 PMC2211528

[19] Strotmann R, Harteneck C, Nunnenmacher K, Schultz G, Plant TD. OTRPC4, a nonselective cation channel that confers sensitivity to extracellular osmolarity. Nat Cell Biol. 2000;2:695–702. 11025659 10.1038/35036318

[20] Liedtke W, Tobin DM, Bargmann CI, Friedman JM. Mammalian TRPV4 (VR-OAC) directs behavioral responses to osmotic and mechanical stimuli in Caenorhabditis elegans. Proc Natl Acad Sci U S A. 2003;100 Suppl 2:14531–6. 14581619 10.1073/pnas.2235619100 PMC304114

[21] Vollrath MA, Kwan KY, Corey DP. The micromachinery of mechanotransduction in hair cells. Annu Rev Neurosci. 2007;30:339–65. 17428178 10.1146/annurev.neuro.29.051605.112917 PMC2865174

[22] Karashima Y, Prenen J, Meseguer V, Owsianik G, Voets T, Nilius B. Modulation of the transient receptor potential channel TRPA1 by phosphatidylinositol 4,5-biphosphate manipulators. Pflugers Arch. 2008;457:77–89. 10.1007/s00424-008-0493-6 18461353

[23] Hill K, Schaefer M. TRPA1 is differentially modulated by the amphipathic molecules trinitrophenol and chlorpromazine. J Biol Chem. 2007;282:7145–53. 17218316 10.1074/jbc.M609600200

[24] Meseguer V, Alpizar YA, Luis E, Tajada S, Denlinger B, Fajardo O, et al TRPA1 channels mediate acute neurogenic inflammation and pain produced by bacterial endotoxins. Nat Commun. 2014;5:3125 10.1038/ncomms4125 24445575 PMC3905718

[25] Engel MA, Leffler A, Niedermirtl F, Babes A, Zimmermann K, Filipović MR, et al TRPA1 and substance P mediate colitis in mice. Gastroenterology. 2011;141:1346–58. 10.1053/j.gastro.2011.07.002 21763243

[26] Kwan KY, Allchorne AJ, Vollrath MA, Christensen AP, Zhang DS, Woolf CJ, et al TRPA1 contributes to cold, mechanical and chemical nociception but is not essential for hair-cell transduction. Neuron. 2006;50:277–89. 16630838 10.1016/j.neuron.2006.03.042

[27] Liedtke W, Friedman JM. Abnormal osmotic regulation in trpv4-/- mice. Proc Natl Acad Sci U S A. 2003;100:13698–703. 14581612 10.1073/pnas.1735416100 PMC263876

[28] Dhaka A, Murray AN, Mathur J, Earley TJ, Petrus MJ, Patapoutian A. TRPM8 is required for cold sensation in mice. Neuron. 2007;54:371–8. 17481391 10.1016/j.neuron.2007.02.024

[29] Bretag AH. Synthetic interstitial fluid for isolated mammalian tissue. Life Sci. 1969;8:319–29. 5781321 10.1016/0024-3205(69)90283-5

[30] Roza C, Reeh PW. Substance P, calcitonin gene related peptide and PGE2 co-released from the mouse colon: a new model to study nociceptive and inflammatory responses in viscera, in vitro. Pain. 2001;93:213–9. 11514080 10.1016/S0304-3959(01)00318-9

[31] Christianson JA, Gebhart GF. Assessment of colon sensitivity by luminal distension in mice. Nature Protocols. 2007;2:2624–31. 17948005 10.1038/nprot.2007.392

[32] Caceres AI, Brackmann M, Elia MD, Bessac BF, del Camino D, D'Amours M, et al A sensory neuronal ion channel essential for airway inflammation and hyperreactivity in asthma. Proc Natl Acad Sci U S A. 2009;106:9099–104. 10.1073/pnas.0900591106 19458046 PMC2684498

[33] Trevisani M, Siemens J, Materazzi S, Bautista DM, Nassini R, Campi B, et al 4-Hydroxynonenal, an endogenous aldehyde, causes pain and neurogenic inflammation through activation of the irritant receptor TRPA1. Proc Natl Acad Sci U S A. 2007;104:13519–24. 17684094 10.1073/pnas.0705923104 PMC1948902

[34] Winget JM, Pan YH, Bahnson BJ. The interfacial binding surface of phospholipase A2s. Biochim Biophys Acta. 2006;1761:1260–9. 16962825 10.1016/j.bbalip.2006.08.002

[35] Pedersen SF, Poulsen KA, Lambert IH. Roles of phospholipase A2 isoforms in swelling- and melittin-induced arachidonic acid release and taurine efflux in NIH3T3 fibroblasts. Am J Physiol Cell Physiol. 2006;291:C1286–96. 16855215 10.1152/ajpcell.00325.2005

[36] Munaron L. Shuffling the cards in signal transduction: Calcium, arachidonic acid and mechanosensitivity. World J Biol Chem. 2011;2:59–66. 10.4331/wjbc.v2.i4.59 21537474 PMC3083947

[37] Mochizuki T, Sokabe T, Araki I, Fujishita K, Shibasaki K, Uchida K, et al The TRPV4 cation channel mediates stretch-evoked Ca2+ influx and ATP release in primary urothelial cell cultures. J Biol Chem. 2009;284:21257–64. 10.1074/jbc.M109.020206 19531473 PMC2755849

[38] Gao X, Wu L, O'Neil RG. Temperature-modulated diversity of TRPV4 channel gating: activation by physical stresses and phorbol ester derivatives through protein kinase C-dependent and-independent pathways. J Biol Chem. 2003;278:27129–37. 12738791 10.1074/jbc.M302517200

[39] Suzuki M, Mizuno A, Kodaira K, Imai M. Impaired pressure sensation in mice lacking TRPV4. J Biol Chem. 2003;278:22664–8. 12692122 10.1074/jbc.M302561200

[40] Rock MJ, Prenen J, Funari VA, Funari TL, Merriman B, Nelson SF, et al Gain-of-function mutations in TRPV4 cause autosomal dominant brachyolmia. Nat Genet. 2008;40:999–1003. 10.1038/ng.166 18587396 PMC3525077

[41] Bautista DM, Siemens J, Glazer JM, Tsuruda PR, Basbaum AI, Stucky CL, et al The menthol receptor TRPM8 is the principal detector of environmental cold. Nature. 2007;448:204–8. 17538622 10.1038/nature05910

[42] Hosoya T, Matsumoto K, Tashima K, Nakamura H, Fujino H, Murayama T, et al TRPM8 has a key role in experimental colitis-induced visceral hyperalgesia in mice. Neurogastroenterol Motil. 2014;26:1112–21. 10.1111/nmo.12368 24832648

[43] Ramachandran R, Hyun E, Zhao L, Lapointe TK, Chapman K, Hirota C, et al TRPM8 activation attenuates inflammatory responses in mouse models of colitis. Proc Natl Acad Sci U S A. 2013;110:7476–81. 10.1073/pnas.1217431110 23596210 PMC3645521

[44] Story GM, Peier AM, Reeve AJ, Eid SR, Mosbacher J, Hricik TR, et al ANKTM1, a TRP-like channel expressed in nociceptive neurons, is activated by cold temperatures. Cell. 2003;112:819–29. 12654248 10.1016/s0092-8674(03)00158-2

[45] Kim D, Cavanaugh EJ, Simkin D. Inhibition of transient receptor potential A1 channel by phosphatidylinositol-4,5-bisphosphate. Am J Physiol Cell Physiol. 2008;295:92–9. 10.1152/ajpcell.00023.2008 PMC2493561 18495815

[46] Doerner JF, Gisselmann G, Hatt H, Wetzel CH. Transient receptor potential channel A1 is directly gated by calcium ions. J Biol Chem. 2007;282:13180–9. 17353192 10.1074/jbc.M607849200

[47] Xiao B, Dubin AE, Bursulaya B, Viswanath V, Jegla TJ, Patapoutian A. Identification of transmembrane domain 5 as a critical molecular determinant of menthol sensitivity in mammalian TRPA1 channels. J Neurosci. 2008;28:9640–51. 10.1523/JNEUROSCI.2772-08.2008 18815250 PMC2678945

[48] Kühn FJP, Kühn C, Lückhoff A. Inhibition of TRPM8 by icilin distinct from desensitization induced by menthol and menthol derivatives. J Biol Chem. 2009;284:4102–11. 10.1074/jbc.M806651200 19095656

[49] Wrigley PJ, Jeong HJ, Vaughan CW. Primary afferents with TRPM8 and TRPA1 profiles target distinct subpopulations of rat superficial dorsal horn neurones. Br J Pharmacol. 2009;157:371–80. 10.1111/j.1476-5381.2009.00167.x 19371346 PMC2707984

[50] Proudfoot CJ, Garry EM, Cottrell DF, Rosie R, Anderson H, Robertson DC, et al Analgesia mediated by the TRPM8 cold receptor in chronic neuropathic pain. Curr Biol. 2006;16:1591–605. 16920620 10.1016/j.cub.2006.07.061

[51] Liu B, Fan L, Balakrishna S, Sui A, Morris JB, Jordt SE. TRPM8 is the principal mediator of menthol-induced analgesia of acute and inflammatory pain. Pain. 2013;154:2169–77. 10.1016/j.pain.2013.06.043 23820004 PMC3778045

[52] Davidson JB, O'Grady G, Arkwright JW, Zarate N, Scott SM, Pullan AJ, et al Anatomical registration and three-dimensional visualization of low and high-resolution pan-colonic manometry recordings. Neurogastroenterol Motil. 2011;23:387–90. 10.1111/j.1365-2982.2010.01651.x 21199536 PMC3080460

[53] Banke TG. The dilated TRPA1 channel pore state is blocked by amiloride and analogues. Brain Res. 2011;1381:21–30. 10.1016/j.brainres.2011.01.021 21241666

[54] Poole DP, Pelayo JC, Cattaruzza F, Kuo YM, Gai G, Chiu JV, et al Transient receptor potential ankyrin 1 is expressed by inhibitory motoneurons of the mouse intestine. Gastroenterology. 2011;141:565–75. 10.1053/j.gastro.2011.04.049 21689654

[55] D'Aldebert E, Cenac N, Rousset P, Martin L, Rolland C, Chapman K, et al Transient receptor potential vanilloid 4 activated inflammatory signals by intestinal epithelial cells and colitis in mice. Gastroenterology. 2011;140:275–85. 10.1053/j.gastro.2010.09.045 20888819

[56] Coste B, Xiao B, Santos JS, Syeda R, Grandl J, Spencer KS, et al Piezo proteins are pore-forming subunits of mechanically activated channels. Nature. 2012;483:176–81. 10.1038/nature10812 22343900 PMC3297710

